# Ju Re Ba Du therapy for Postherpetic neuralgia: A protocol for systematic review and meta-analysis

**DOI:** 10.1097/MD.0000000000022992

**Published:** 2020-10-30

**Authors:** Shijie Huang, Zhengqi Pan, Zimeng Li, Xinyun Zhu, Tingting Ma, Jie Wu

**Affiliations:** a Acupuncture and Moxibustion School Chengdu University of Traditional Chinese Medicine; b Hospital of Chengdu University of Traditional Chinese Medicine, Chengdu, Sichuan, China.

**Keywords:** Ju Re Ba Du therapy, meta-analysis and systematic review, Postherpetic neuralgia, protocol

## Abstract

**Introduction:**

Postherpetic neuralgia (PHN) is one of the most common types of chronic neuropathic pain, which seriously affects quality of the life because of pain severity and poor response to the currently available treatments. Ju Re Ba Du therapy as a form of acupuncture therapy which is proved to be effective in RCTs and very suitable for patients, has been used in Postherpetic neuralgia in patients for a long time, therefore a systematic review is necessary to provide available evidence for further study.

**Methods and analysis:**

The following databases will be searched from their inception to October 2020: Electronic database includes PubMed, Embase, Cochrane Library, Web of Science, Nature, Science online, VIP medicine information, and CNKI (China National Knowledge Infrastructure). Primary outcome: pain intensity assessed on a visual analogue scale (VAS); Additional outcomes:

1. Quality of life assessed by the 36-Item Short-Form Health Survey (SF-36);
2. Self-Rating Anxiety Scale (SAS);
3. Self-Rating Depression Scale (SDS);
4. Sleep quality measured by the Pittsburgh Sleep Quality Index (PSQI).

Data will be extracted by two researchers independently, risk of bias of the meta-analysis will be evaluated based on the Cochrane Handbook for Systematic Reviews of Interventions. All data analysis will be conducted by data statistics software Review Manager V.5.3. and Stata V.12.0.

**Results:**

The results of this study will systematically evaluate the effectiveness and safety of Ju Re Ba Du therapy intervention for people with Postherpetic neuralgia.

**Conclusion:**

The systematic review of this study will summarize the current published evidence of Ju Re Ba Du therapy for the treatment of Postherpetic neuralgia, which can further guide the promotion and application of it.

**Ethics and dissemination:**

This study is a systematic review, the outcomes are based on the published evidence, so examination and agreement by the ethics committee are not required in this study. We intend to publish the study results in a journal or conference presentations.

**OSF registration number:**

September 29, 2020 osf.io/r6y9b. (https://osf.io/r6y9b)

## 1 Introduction

Herpes zoster (HZ) is a common and frequently-occurring disease among middle-aged and elderly people. The global incidence is increasing year by year, reaching 25% to 30%, and tends to increase with age. The average age of onset of HZ is 51.7 years old.^[1]^ In Europe, the incidence is 7 to 8 per 1,000 in groups aged≥50 years, and 10 per 1,000 in groups aged≥80 years.^[2]^ There is no large-scale epidemiological investigation on HZ in China, but according to the results of some small-scale investigations, the incidence of HZ in China is close to that of other countries.^[3,4]^ As the most serious complication of HZ, the incidence of Postherpetic neuralgia (PHN) also increases with age,^[5]^ and its pain symptoms are obvious, lasting from several months to several years, which seriously affects the quality of life of patients and brings serious economic burden and public health problems to the society.

Postherpetic neuralgia (PHN) is one of the most common types of chronic neuropathic pain, which seriously affects quality of the life because of pain severity and poor response to the currently available treatments. The main strategies for PHN management are medication and invasive interventional therapies; however, these approaches have many adverse effects, so it is important to find another effective and safe treatment for PHN.^[6]^ Neuropathic pain, such as postherpetic neuralgia, is a chronic disease like hypertension and diabetes, and requires long-term treatment to improve or control symptoms.^[7]^

There is no consensus on the specific treatment drugs and diagnosis. At present, the clinical treatment is mostly empirical treatment, mainly including physical therapy, drug treatment, traditional Chinese medicine treatment, nerve block and so on,^[8]^ but the therapeutic effect is not satisfactory. Scholars at home and abroad have carried out many studies on the treatment, and the exploration has attracted the attention from many disciplines.

In recent years, acupuncture and moxibustion has been widely used in clinical and experimental studies of PHN, and its effectiveness has been fully proved. As a form of acupuncture and moxibustion, Ju Re Ba Du therapy has been used to relieve symptoms in Postherpetic neuralgia patients, but its effectiveness and safety have not yet reached a definitive conclusion.^[9]^ Therefore, this research intends to adopt the method of system valuation and meta-analysis of the Ju Re Ba Du therapy for Postherpetic neuralgia to evaluate the efficacy and safety.

## 2 Methods

### 2.1 Study registration

The protocol of the systematic review has been registered.

Registration: Open Science Fra network (OSF) registration number: September 29, 2020 osf.io/r6y9b. (https://osf.io/r6y9b). This systematic review protocol will be conducted and reported strictly according to Preferred Reporting Items for Systematic Reviews and Meta-Analyses (PRISMA)^[10]^ statement guidelines, and the important protocol amendments will be documented in the full review.

### 2.2 Inclusion and exclusion criteria for study selection

#### 2.2.1 Inclusion criteria

Inclusion criteria are all randomized controlled trials (RCTs), which main treatment of Postherpetic neuralgia is Ju Re Ba Du therapy. The language of the trials to be included only Chinese or English.

#### 2.2.2 Exclusion criteria. Following studies will be excluded:

1. Repeated publications
2. Review of literature and cases
3. Animal studies
4. Incomplete literature
5. Non-randomized controlled trials

### 2.3 Types of participants

The types of subjects included patients diagnosed with Postherpetic neuralgia, regardless of their degree and possible complications. All patients were treated with Ju Re Ba Du therapy.

### 2.4 Interventions and controls

Interventions included treatment with Ju Re Ba Du therapy. The control group only received conventional western medicine treatment. The routine treatment of each RCT may not be identical, but the use of Ju Re Ba Du therapy is the only difference between intervention and control.

### 2.5 Types of outcome measures

#### 2.5.1 Main outcomes

1. Pain intensity assessed on a visual analogue scale (VAS)

#### 2.5.2 Additional outcomes

1. Quality of life assessed by the 36-Item Short-Form Health Survey (SF-36);
2. Self-Rating Anxiety Scale (SAS)
3. Self-Rating Depression Scale (SDS)
4. Sleep quality measured by the Pittsburgh Sleep Quality Index (PSQI).

### 2.6 Search methods

#### 2.6.1 Search resources

**Figure 1:**
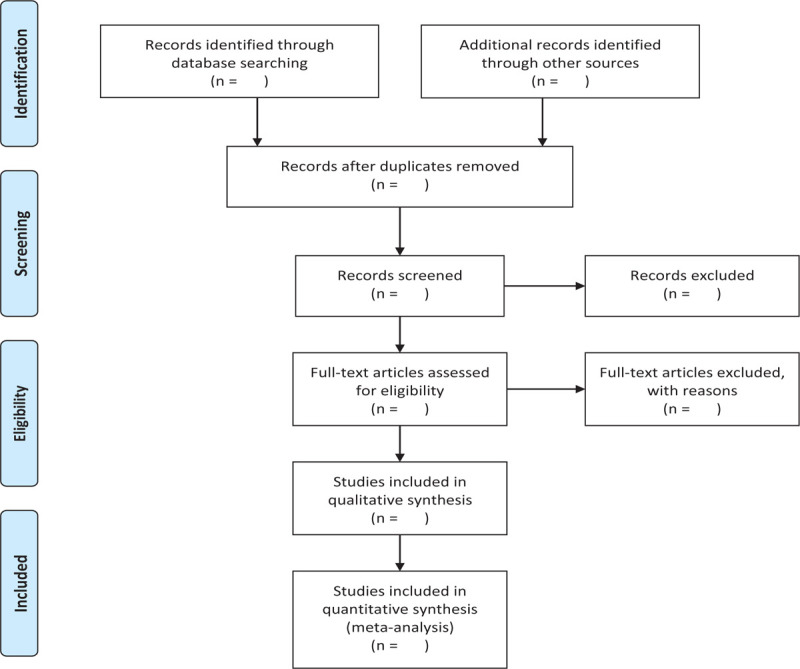
The research flowchart. This figure shows the Identification, Screening, Eligibility and Included when we searching articles.

We will search the following electronic databases from their inception to October 2020: Electronic database includes PubMed, Embase, Cochrane Library, Chinese Biomedical Database, VIP medicine information, and CNKI (China National Knowledge Infrastructure). (Fig. 1.) The research flowchart.

#### 2.6.2 Search strategies

The following MeSH terms and their combinations will be searched: (1) Ju Re Ba Du therapy; (2) RCT OR RCTs; (3) Postherpetic neuralgia. (Table 1.) The research strategy.

**Table 1:**
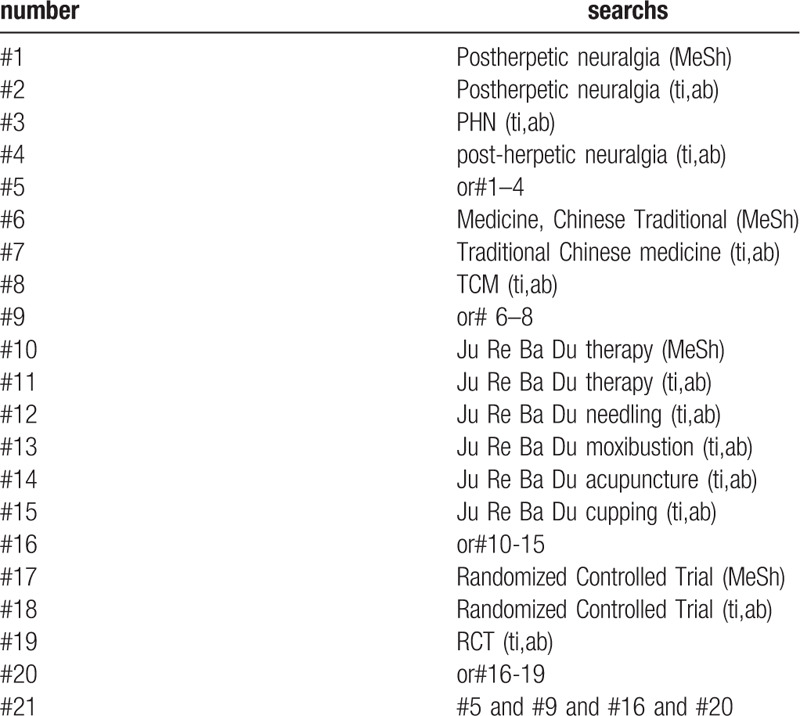
The research strategy. Table 1 Search strategy sample of PubMed.

### 2.7 Data collection and analysis

#### 2.7.1 Studies selection

There will be two researchers (SH and ZP) carry out the selection of research literature independently using Note-Express software. We will first make the preliminary selection by screening titles and abstracts. Secondly, we will download full text of the relevant studies for further selection according to the inclusion criteria. If there is any different opinion, two researchers will discuss and reach an agreement. If a consensus could not be reached, there will be a third researcher (JW) who make the final decision. The details of selection process will be displayed in the PRISMA flow chart.

#### 2.7.2 Data extraction

Two researchers (SH and ZP) will read all the included text in full, and independently extract the following information:

(1) general information, including trial name and registration information;
(2) trial characteristic, including trial design, location, setting, and inclusion/exclusion criteria;
(3) the characteristics of the participants, including age, race / ethnicity, course of illness, etc.
(4) details of intervention, including acupoints, time of intervention, course of treatment, time of single treatment, etc.
(5) details of comparison interventions;
(6) out- comes as described under type of outcome measure section.

If we couldn’t reach an agreement, a third researcher (JW) would make the final decision. One researcher (ZL) would contact the corresponding author by telephone or e-mail for more information when the reported data were insufficient or ambiguous.

#### 2.7.3 Assessment of risk of bias

All the included studies will be evaluated based on the guidelines of Cochrane Handbook for Systematic Reviews of Interventions.^[11]^ The quality of each trial will categorized into ‘low’, ‘unclear’, or ‘high’ risk of bias according to the following items: adequacy of generation of the allocation sequence, allocation concealment, blinding of participants and personal, blinding of outcome assessors, incomplete outcome data, selected reporting the results and other sources of bias (such as comparable baseline characteristic, inclusion and exclusion criteria).

#### 2.7.4 Assessment of reporting biases

Reporting biases and small-study effects will be detected by funnel plot and Egger test if there are 10 more studies included in this Meta- analysis. For Egger test, *P* value of < 0.10 was considered to indicate the exist of reporting biases and small study effects.

#### 2.7.5 Data analysis

We used Revman 5.3 software provided by the Cochrane collaboration to analyze the data. Binary outcomes will be summarized using risk ratio (RR) with 95% confidence interval (CI) for relative effect. Continuous outcomes will be summarized by using weighted mean difference (WMD) with 95% CI. We will use random-effect model (REM) for meta-analysis in this review according to research recommendations.^[12]^

Statistical heterogeneity will be assessed by X^2^ and I^2^ statistical tests. Where *P* value ≥0.1and I^2^ ≤50%, there is no obvious statistical heterogeneity among the studies. On the contrary, where *P* value < 0.1or I^2^ > 50% indicates a considerable heterogeneity. Meta-analysis will be performed when the statistical heterogeneity is acceptable (*P* value ≥0.1and I^2^ ≤50%), otherwise, subgroup analysis will be applied to explore the influence of potential factors on the outcome measures. We will conduct sensitivity analyses by omitting studies one by one in order to probe the impact of an individual study. If a meta-analysis cannot be performed, we will conduct descriptive analysis instead.

#### 2.7.6 Patient and public involvement

This is a meta-analysis study based on previously published data, so patient and public involvement will not be included in this study.

#### 2.7.7 Ethics and dissemination

Ethical approval will not be required as this is a protocol for systematic review and meta-analysis. The findings of this study will be disseminated to a peer-reviewed journal and presented at a relevant conference.

#### 2.7.8 Evidence assessed

The quality of evidence for this study will be assessed by “Grades of Recommendations Assessment, Development and Evaluation (GRADE) standard established by the World Health Organization and international organizations.^[13]^ To achieve transparency and simplification, the quality of evidence is divided into 4 levels in GRADE system: high, medium, low and very low. We will employ GRADE profiler 3.2 for analysis.^[14]^

## 3 Discussion

Zoster (HZ) is caused by the human body being infected with Varicella Zoster virus (VZV). The virus is latent in the neurons of the ganglion, and as the host grows older or the immune function is reduced, the virus is reactivated. The main clinical manifestations were ganglionic clustered red acromial herpes and neuralgia.^[15]^ Studies have shown that the global incidence of HZ increases year by year.^[16,17]^ Postherpetic neuralgia (PHN) is the most serious complications in HZ, is characterized by severe pain, its incidence of 5% to 30%, when patients older than 85 years old was 50%, and the higher incidence of pain and sustainable for several months to several years, the serious influence the patient's quality of life, brings the serious economic burden to society, caused a public health problem.^[18,19]^

Acupuncture and moxibustion is a common intervention method of HZ and PHN. Meta-analysis showed that acupuncture and moxibustion had better efficacy in terms of cure rate, removal time and pain improvement, no matter in the acute stage, crusting stage or post-neuralgia stage.^[20,21]^ 2014 book “evidence-based acupuncture clinical practice guidelines herpes zoster (revised edition)” is now the disease only acupuncture clinical scheme, although the guide lists the different recommended level of acupuncture and moxibustion treatment, but the plan is not fixed, clinicians may, in accordance with the personal experience a bias when choosing different treatment methods, which can lead to clinical curative effect is not stable, differences of health economics. Therefore, it is necessary to form a more unified and effective acupuncture intervention program through the study of optimized program, so as to facilitate clinical promotion. The treatment regimens adopted in this study not only clearly list different treatment methods in different periods, but also specify the treatment sequence and treatment duration. Therefore, the optimized regimen can reduce the bias in the selection of treatment methods, narrow the differences in health economics, and have more clinical promotion value.

HZ and PHN's pathogenesis is complex, involving many aspects, inflammatory corpuscle is an important component of the innate immune system, including NLRP3 inflammatory corpuscle by nucleotide combination structure of oligomerization domain (nucleotide binding oligomerization domain, NOD) members of the family of receptors NLRP3, protein ASC and cohesion in radix asparagi protease precursor of caspase - 1, ASC for linking NLRP3 and scaffold protein procaspase - 1,Procaspase-1, on the other hand, can shear the proinflammatory factor IL-1, the precursor of IL-18, and cause the proinflammatory factor to mature and release, thus triggering the inflammatory response.^[22]^ Th17 has pro-inflammatory properties, which can stimulate the synthesis of various inflammatory factors and induce local reactions. Treg cells are T cells with active inhibitory effect and anti-inflammatory properties. Th17/IL-17 plays an important role in the body's immune defense and is a key factor for the occurrence and development of autoimmune diseases. However, silencing NLRP3 can inhibit the expression of inflammatory cytokines IL-1, IL-23 and chemokine CXCL1, thereby upregulating the Treg/Th17 cell ratio.^[23]^ Studies have shown that NLRP3 inflammasome can regulate the activation of spinal microglia cells and participate in the neuropathic pain process in rats.^[24,25]^ HZ/PHN pain also belongs to neuropathic pain. At present, there are few studies on HZ/PHN and NLRP3 inflamriome, and only 1 paper in 2011 showed that VZV virus can trigger the formation of NLRP3 inflamriome.^[26]^ Pain, as a symptom that most affects the quality of life in Patients with HZ/PHN, is closely related to the SP/NK-1R axis. SP is widely distributed in various tissues of the body and is an important neurokinin related to the regulation of harmful information. It plays an important role in pain transmission, inflammation, immune regulation and other aspects.^[27,28]^ It often binds to nK-R1 receptors, thus causing cell excitement, increased sensitivity, and induced immune function,^[29]^ stimulating the secretion of inflammatory factors, increasing vascular permeability, and vascular dilation, thus causing pain.^[30–32]^ Therefore, it is found that NLRP3 inflammasome may play a role in acupuncture treatment of HZ/PHN.

Ju Re Ba Du therapy for postherpetic neuralgia can significantly improve the pain symptoms of patients with significant clinical effects, and is worthy of further promotion and application in clinical practice.^[33]^

In summary, this systematic review will be an evaluation of the efficacy and safety of Ju Re Ba Du of postherpetic neuralgia. On this basis, a better processing method can be established to provide a reliable basis for its wide application.

## Author contributions

**Conceptualization:** Shijie Huang, Zhengqi Pan.

**Data curation:** Shijie Huang, Zhengqi Pan, Tingting Ma.

**Formal analysis:** Shijie Huang, Zhengqi Pan.

**Investigation:** Jie Wu, Zimeng Li.

**Methodology:** Jie Wu.

**Project administration:** Shijie Huang.

**Resources:** Zimeng Li.

**Software:** Xinyun Zhu, Tingting Ma.

**Supervision:** Jie Wu.

**Validation:** Xinyun Zhu.

**Writing – original draft:** Shijie Huang, Zhengqi Pan.

**Writing – review & editing:** Shijie Huang, Zhengqi Pan.
